# The Relationship of Platelets With the Clinical Manifestations and Serologic Markers in Systemic Lupus Erythematosus: A Single‐Center Retrospective Study

**DOI:** 10.1002/iid3.70201

**Published:** 2025-04-23

**Authors:** Jinlu Ma, Lin Zhang, Mengxue Yan, Zhichun Liu, Leixi Xue

**Affiliations:** ^1^ Department of Rheumatology and Immunology Songjiang Hospital Affiliated to Shanghai Jiao Tong University School of Medicine Shanghai China; ^2^ Department of Rheumatology and Immunology the Second Affiliated Hospital of Soochow University Suzhou China

**Keywords:** clinical features, complement, platelet count, systemic lupus erythematosus, thrombocytopenia

## Abstract

**Background:**

Thrombocytopenia is a common clinical manifestation of Systemic lupus erythematosus (SLE), and platelets may play a central role in the pathogenesis of immune‐mediated inflammatory diseases. The study aimed to investigate the relationship between platelet count and clinical manifestations and serologic markers in systemic lupus erythematosus (SLE).

**Methods:**

This single‐center retrospective study extracted demographic data, blood cell counts, complement (C) levels, autoantibody profiles, and clinical presentation information from the electronic medical records of patients with SLE. The SLE Disease Activity Index 2000 (SLEDAI 2000) score was calculated, and Spearman's correlation coefficient was used to evaluate the correlation between platelet count and other variables.

**Results:**

A total of 418 patients with SLE were included in the study. The platelet count was correlated with hemoglobin, complement 3 (C3), and C4 levels; leukocyte, neutrophil, and lymphocyte counts; and the SLEDAI 2000 score. In patients with SLE with thrombocytopenia, the platelet count was associated with the hemoglobin level and a positive direct Coombs' test. The platelet count in patients with SLE without thrombocytopenia was significantly lower compared with the healthy controls, and the platelet count in this group was correlated with C3 and C4 levels, as well as the leukocyte, neutrophil, and lymphocyte counts. The disease characteristics of patients with SLE with thrombocytopenia differed from those of patients with SLE without thrombocytopenia, whereas the clinical features were essentially the same between patients with mild to moderate SLE‐associated thrombocytopenia and patients with severe SLE‐associated thrombocytopenia.

**Conclusion:**

Patients with SLE can be categorized into two groups with different clinical features based on the presence or absence of thrombocytopenia. Furthermore, the platelet count correlates with other blood cell counts and complement levels, particularly in patients with SLE without thrombocytopenia.

## Graphical abstract

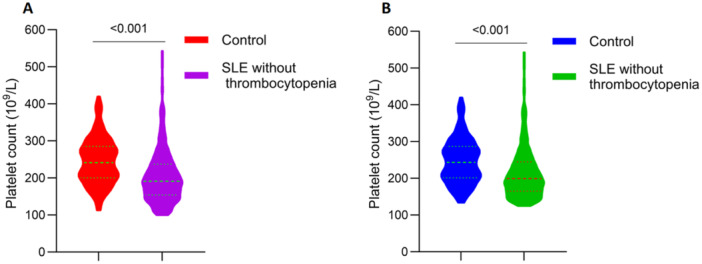

## 1 Introduction

Systemic lupus erythematosus (SLE) is a systemic inflammatory autoimmune disease with highly heterogeneous symptoms and manifestations [1]. SLE pathogenesis involves genes, environmental factors, estrogen levels, and an abnormally activated immune system, but the exact mechanisms remain unclear [1].

Thrombocytopenia is a common clinical manifestation of SLE, with a prevalence of 10%–40% [2]. The risk factors for thrombocytopenia in patients with SLE have been widely explored because thrombocytopenia increases the risk of mortality [3, 4, 5]. Treatment for thrombocytopenia has also been adequately investigated, and many studies have explored treatment options for refractory thrombocytopenia [6, 7].

In addition to their established role in primary hemostasis, there is now evidence that platelets are an integral part of the immune system, playing a central role in the pathogenesis of immune‐mediated inflammatory diseases [8]. Immune complexes and viral infections induce platelet activation via receptors on the platelet surface, such as Fcγ receptor IIA (FcγRIIA) and Toll‐like receptor (TLR) 7 [9, 10, 11]. Activated platelets release dense granules and α‐granules that further amplify platelet activation via adenosine triphosphate or adenosine diphosphate and complement (C) release [12]. Glycoproteins, such as CD40L and P‐selectin, relocate from cytoplasmic granules to the surface of activated platelets, where they interact with immune cells [9, 13]. Extracellular vesicles produced by activated platelets contain many molecules that diffuse platelet components into body fluids and tissues normally inaccessible to platelets, such as the lymphatic system, as was demonstrated in a mouse model of inflammatory arthritis [14, 15].

Besides the risk of bleeding and death due to thrombocytopenia, the clinical significance of platelets in SLE remains unelucidated. Thus, this study aimed to investigate the relationship between platelet count and clinical manifestations and serologic markers in SLE to provide clinical evidence of platelet involvement in SLE pathogenesis.

## 2 Methods

### 2.1 Study Design

This single‐center retrospective study was conducted in the Department of Rheumatology and Immunology at The Second Affiliated Hospital of Soochow University, China. We used inpatient medical records from June 2009 to December 2022 to consecutively enroll patients with SLE in our study. Patients with SLE were included in the thrombocytopenia group if they had SLE‐induced thrombocytopenia during their hospitalisation at our hospital, whereas patients without thrombocytopenia had to satisfy that they had not developed SLE‐induced thrombocytopenia at any time during the course of their illness, including before their hospitalisation at our hospital and during subsequent follow‐up visits. The control group comprised healthy individuals from the physical examination center. This study followed the STROBE guidelines, and was conducted in accordance with the World Medical Association Declaration of Helsinki and was approved by the Ethics Review Committee of The Second Affiliated Hospital of Soochow University (Approval No. JD‐HG‐2023‐105). Due to its retrospective nature, informed consent was waived by the Human Ethics Review Committee.

SLE diagnosis was made according to one of the following three criteria: the American College of Rheumatology (ACR) 1997 revised criteria, the 2012 Systemic Lupus International Collaborating Clinics classification criteria, or the 2019 European League Against Rheumatism/ACR classification criteria. Patients with SLE were excluded if they had one or more of the following conditions: (1) anti‐phospholipid antibody syndrome, (2) thrombotic microangiopathy, (3) hepatosplenic disease (portal hypertension, cirrhosis, and splenomegaly, etc.), (4) primary hematological disease (lymphoma, myelodysplastic syndrome, and leukemia, etc.), (5) drug‐induced cytopenia (thrombocytopenia, leukopenia and myelosuppression), (6) pregnancy, or (7) severe infectious diseases.

### 2.2 Data Collection

The electronic medical records of all study subjects were used to extract data on demographics and clinical presentation, as well as laboratory test results (blood cell counts, C‐reactive protein, erythrocyte sedimentation rate, complement levels, and autoantibody profiles). Data for patients with thrombocytopenia were obtained from the time of the patient's first episode of thrombocytopenia during their hospitalisation at our hospital, whereas data for patients without thrombocytopenia were obtained from the time of the patient's first hospitalisation at our hospital. Based on the data collected above, the SLE Disease Activity Index 2000 (SLEDAI 2000) score was calculated, and SLEDAI 2000 scores excluding thrombocytopenia scores were defined as modified SLEDAI 2000 (mSLEDAI 2000) scores. Only demographic data and platelet count results were collected in the healthy control group.

Thrombocytopenia was defined as a platelet count < 100 × 10^9^/L, leukopenia as a leukocyte count < 3.0 × 10^9^/L, neutropenia as a neutrophil count < 1.5 × 10^9^/L, lymphopenia as a lymphocyte count < 0.8 × 10^9^/L, and anemia as a hemoglobin level < 110 g/L or < 120 g/L for females and males, respectively, regardless of etiology.

### 2.3 Data Analysis

Categorical variables were expressed as absolute frequencies and percentages, and differences between groups were assessed using chi‐square test or Fisher exact test. Continuous variables were expressed as mean ± SD or median and the 25th–75th percentile (*P*_25_–*P*_75_) according to Shapiro–Wilk test. Normally distributed variables were analyzed using Student *t*‐test and skewed variables using Mann–Whitney U test. Spearman correlation coefficient was used to calculate the correlation between platelet count and other variables. The significance level was set at *p* < 0.05. All statistical analyses were performed using IBM SPSS Statistics for Windows v21.0 (IBM Corp., Armonk, NY).

## 3 Results

### 3.1 Baseline Clinical Characteristics

Our study included 418 patients with SLE (female, 89.5%) with a median disease duration of 12.0 (2.0–72.0) months and a median age of 35.0 (27.0–48.0) years (Supporting Information S1: Table S1). Leukopenia, neutropenia, lymphopenia, thrombocytopenia, and lupus nephritis were observed in 21.5% (90), 9.6% (40), 36.6% (153), 26.1% (109), and 63.2% (264) of the patients with SLE, respectively. The median SLEDAI 2000 score was 13.0 (8.0–19.0).

### 3.2 Relationship Between Platelet Count and Clinical Indicators in Patients With SLE

We used Spearman correlation test to assess the correlation between platelet count and clinical indicators, including clinical presentation, serological indicators, and SLEDAI 2000 score, in patients with SLE at the time of thrombocytopenia (Table 1). The results revealed that platelet count was correlated with leukocyte, neutrophil, and lymphocyte counts; hemoglobin, C3, and C4 levels; and the SLEDAI 2000 score. There was no correlation between platelet count and any other indicators.

**Table 1:** Correlation of platelet count with other indices in patients with SLE using Spearman correlation test.

	All patients		With thrombocytopenia		Without thrombocytopenia	
	*Rho*	*p*	*Rho*	*p*	*Rho*	*p*
Leukocyte count	0.354	< 0.001	−0.111	0.249	0.398	< 0.001
Neutrophil count	0.317	< 0.001	−0.125	0.195	0.395	< 0.001
Lymphocyte count	0.257	< 0.001	−0.114	0.239	0.186	0.001
Hemoglobin	0.246	< 0.001	0.209	0.029	0.050	0.380
C‐reactive protein	0.059	0.227	0.021	0.826	0.064	0.264
Erythrocyte sedimentation rate	0.061	0.222	0.068	0.489	0.080	0.169
Anti‐dsDNA	0.007	0.883	0.009	0.923	−0.054	0.346
Antiphospholipid antibodies	−0.036	0.585	−0.102	0.407	−0.106	0.177
Anticardiolipin	−0.016	0.812	0.150	0.659	−0.082	0.297
Anti–β2‐glycoprotein I	−0.029	0.664	−0.535	0.111	−0.011	0.888
Direct Coombs' test	−0.044	0.627	−0.296	0.041	0.147	0.206
Complement 3	0.272	< 0.001	−0.001	0.995	0.233	< 0.001
Complement 4	0.209	< 0.001	−0.019	0.846	0.206	< 0.001
Fever	−0.082	0.095	0.118	0.220	0.011	0.844
Rash	−0.046	0.352	0.034	0.728	−0.057	0.318
Alopecia	−0.010	0.839	−0.036	0.711	−0.073	0.198
Oral ulcers	−0.029	0.551	0.159	0.098	−0.013	0.823
Arthritis	0.024	0.623	0.036	0.709	−0.020	0.728
Myositis	−0.021	0.668	−0.029	0.761	−0.029	0.614
Vasculitis	−0.061	0.215	−0.030	0.760	−0.095	0.096
Pleurisy	0.044	0.365	0.051	0.601	0.083	0.146
Pericarditis	0.014	0.768	−0.039	0.689	0.048	0.400
Lupus nephritis	−0.046	0.349	−0.055	0.572	0.014	0.801
Neurologic involvement	−0.074	0.131	0.018	0.849	0.028	0.628
Cardiac involvement	0.028	0.571	0.002	0.987	0.087	0.125
Interstitial lung disease	0.010	0.841	−0.176	0.067	0.050	0.385
SLEDAI 2000 score	−0.180	< 0.001	−0.016	0.866	−0.087	0.134

Abbreviations: SLE, systemic lupus erythematosus; SLEDAI 2000, systemic lupus erythematosus disease activity index 2000.

Thrombocytopenia is associated with SLE disease activity [16]. Therefore, we further categorized patients with SLE into two groups: those with thrombocytopenia and those without thrombocytopenia. In patients with SLE with thrombocytopenia, platelet count was associated with hemoglobin levels and a positive direct Coombs' test, but there was no correlation with other parameters, including SLEDAI 2000 score and C3 and C4 levels (Table 1). In patients with SLE without thrombocytopenia, platelet count was correlated with leukocyte, neutrophil, and lymphocyte counts, as well as C3 and C4 levels, but not with hemoglobin levels, a positive direct Coombs' test, or the SLEDAI 2000 score (Table 1).

### 3.3 Comparison of Platelet Count Between Patients With SLE Without Thrombocytopenia and Healthy Controls

**Figure 1:**
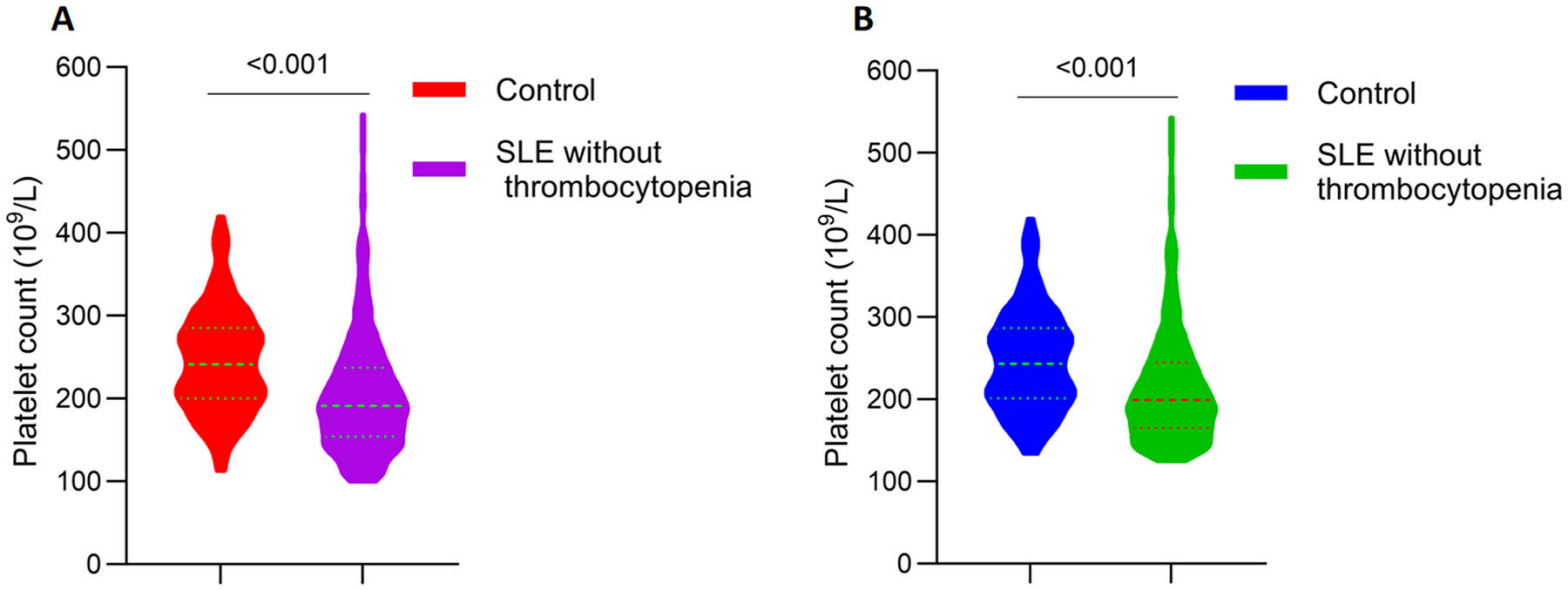
Comparison of platelet count between patients with SLE without thrombocytopenia and healthy controls. (A) Thrombocytopenia was defined as a platelet count of < 100 × 10^9^/L; (B) Thrombocytopenia was defined as a platelet count of < 125 × 10^9^/L.

Because platelet counts in patients with SLE without thrombocytopenia were correlated with C3 and C4, which are serum indicators of SLE disease activity, we speculated that platelet counts might also be decreased in patients with SLE without thrombocytopenia. Therefore, we recruited 151 healthy age‐ and gender‐matched controls and compared their platelet counts with those of patients with SLE without thrombocytopenia. The median platelet count in the healthy controls and in patients with SLE without thrombocytopenia was 241 (200–285) × 10^9^/L and 191 (154–237) × 10^9^/L, respectively, and this difference was statistically significant (*p* < 0.001; Figure 1A). Considering that the lower limit of the normal platelet count range in our centre is 125 × 10^9^/L, we then excluded participants whose platelet count fell between 100 and 125 × 10^9^/L (2 healthy controls and 29 patients with SLE). Subsequent analysis revealed that the platelet count remained significantly higher in the healthy controls compared with patients with SLE without thrombocytopenia (*p* < 0.001; Figure 1B).

### 3.4 Comparison of the Clinical Characteristics Between Patients With SLE With Thrombocytopenia and Patients With SLE Without Thrombocytopenia

Surprisingly, almost opposite results were found in patients with SLE without thrombocytopenia compared with patients with SLE with thrombocytopenia. Therefore, we further compared the clinical characteristics between the two groups (Table 2). Patients with SLE with thrombocytopenia had lower hemoglobin levels and leukocyte, neutrophil, and lymphocyte counts compared with patients with SLE without thrombocytopenia. Furthermore, more patients with SLE with thrombocytopenia exhibited leukopenia, lymphopenia, anemia, and fever compared with patients with SLE without thrombocytopenia. Regarding autoantibodies and disease activity indicators, compared with patients with SLE without thrombocytopenia, patients with SLE with thrombocytopenia had a lower positivity rate for anti‐Ro60 and anti‐Ro52, lower C3 and C4 levels, and higher SLEDAI 2000 scores, even when the thrombocytopenia score was excluded from the SLEDAI 2000 score, suggesting that patients with SLE with thrombocytopenia had higher disease activity compared with patients with SLE without thrombocytopenia. Thus, patients with SLE with thrombocytopenia and patients with SLE without thrombocytopenia may represent two distinct groups of patients with SLE that exhibit a high degree of heterogeneity.

**Table 2:** Comparison of the clinical characteristics of patients with SLE with thrombocytopenia and patients with SLE without thrombocytopenia.

Variable	With thrombocytopenia		Without thrombocytopenia		*p*
	Value	*N*	Value	*N*	
Leukocyte count (10^9^/L)	3.6 (2.6–5.4)	109	4.6 (3.3–6.9)	309	< 0.001
Leukopenia, *n* (%)	38 (34.9)	109	52 (16.8)	309	< 0.001
Neutrophil count (10^9^/L)	2.3 (1.7–4.0)	109	2.9 (2.0–4.9)	309	0.002
Neutropenia, *n* (%)	14 (12.8)	109	26 (8.4)	309	0.176
Lymphocyte count (10^9^/L)	0.7 (0.5–1.2)	109	1.0 (0.7–1.5)	309	< 0.001
Lymphopenia, *n* (%)	57 (52.3)	109	96 (31.1)	309	< 0.001
Hemoglobin (g/L)	98.0 (78.0–114.0)	109	112.0 (98–126.5)	309	< 0.001
Anemia^a^, *n* (%)	74 (67.9)	109	140 (45.3)	309	< 0.001
C‐reactive protein (mg/L)	6.0 (4.9–10.3)	109	5.9 (5.3–9.1)	309	0.496
Erythrocyte sedimentation rate (mm/H)	30 (13–51)	106	32 (15–54)	298	0.725
Antinuclear antibody titre	320 (320–1000)	109	1000 (320–1000)	298	0.050
Anti‐dsDNA, *n* (%)	61 (56.0)	109	186 (61.2)	304	0.340
Anti‐Smith, *n* (%)	33 (30.3)	109	107 (35.8)	299	0.300
Anti‐U1RNP, *n* (%)	37 (33.9)	109	131 (44.0)	298	0.069
Anti‐ribosomal P protein, *n* (%)	22 (20.2)	109	75 (25.2)	298	0.296
Anti‐Ro60, *n* (%)	53 (48.6)	109	194 (65.1)	298	0.003
Anti‐SSB, *n* (%)	15 (13.8)	109	50 (16.8)	298	0.462
Anti‐Ro52, *n* (%)	44 (40.4)	109	168 (56.4)	298	0.004
Anti‐centromere protein B, *n* (%)	5 (4.6)	109	6 (2.0)	298	0.283
Antiphospholipid antibodies, *n* (%)	11 (16.2)	68	32 (19.5)	164	0.552
Anticardiolipin	10 (14.7)	68	30 (18.3)	164	0.510
Anti–β2‐glycoprotein I	4 (5.9)	68	9 (5.5)	164	1.000
Direct Coombs' test, n (%)	14 (29.2)	48	18 (23.7)	76	0.497
Complement 3 (g/L)	0.448 (0.300–0.657)	109	0.590 (0.400–0.800)	304	< 0.001
Complement 4 (g/L)	0.080 (0.060–0.137)	109	0.106 (0.078–0.156)	304	0.010
Fever, *n* (%)	36 (33.0)	109	64 (20.7)	309	0.010
Rash, *n* (%)	50 (45.9)	109	134 (43.4)	309	0.650
Alopecia, *n* (%)	32 (29.4)	109	105 (34.0)	309	0.377
Oral ulcers, *n* (%)	16 (14.7)	109	35 (11.3)	309	0.358
Arthritis, *n* (%)	17 (15.6)	109	60 (19.4)	309	0.378
Myositis, *n* (%)	3 (2.8)	109	8 (2.6)	309	1.000
Vasculitis, *n* (%)	21 (19.3)	109	57 (18.5)	309	0.850
Pleurisy, *n* (%)	16 (14.7)	109	44 (14.2)	309	0.910
Pericarditis, *n* (%)	14 (12.8)	109	37 (12.0)	309	0.811
Lupus nephritis, *n* (%)	75 (68.8)	109	189 (61.6)	307	0.177
Neurologic involvement, *n* (%)	13 (11.9)	109	16 (5.2)	309	0.017
Cardiac involvement, *n* (%)	9 (8.3)	109	21 (6.8)	309	0.611
Interstitial lung disease, *n* (%)	6 (5.5)	109	16 (5.2)	309	0.896
SLEDAI 2000 score	16.0 (10.0–24.0)	109	12.0 (8.0–18.0)	301	0.001
mSLEDAI 2000 score	15.0 (9.00–23.00)	109	12.0 (8.0–18.0)	301	0.015

*Note:* Except where indicated otherwise, values are median (*P*_25_, *P*_75_).

Abbreviations: SLE, systemic lupus erythematosus; mSLEDAI 2000, modified systemic lupus erythematosus disease activity index 2000; SLEDAI 2000, systemic lupus erythematosus disease activity index 2000.

^a^ Anemia was defined as a hemoglobin level < 110 g/L for females or < 120 g/L for males, regardless of etiology.

### 3.5 Comparison of Clinical Characteristics of Patients With SLE With Mild to Moderate Thrombocytopenia and Patients With SLE With Severe Thrombocytopenia

Referring to the LUMINA cohort from the United States [17], we categorized mild to moderate thrombocytopenia as 50–100 × 10^9^/L and severe thrombocytopenia as < 50 × 10^9^/L), using a platelet count of 50 × 10^9^/L as the cutoff. No statistically significant differences in clinical presentation, autoantibody profiles, or disease activity were observed between the two groups (Supporting Information S1: Table S2). Then, we repeated the analysis using a platelet count of 30 × 10^9^/L as the cutoff [4]. The results revealed a statistically significant difference between the two groups in terms of interstitial lung disease only (Table 3). Therefore, patients with thrombocytopenia may be a relatively homogeneous group in SLE.

**Table 3:** Comparison of the clinical characteristics of patients with SLE with mild to moderate thrombocytopenia (30–100 × 10^9^/L) and patients with SLE with severe thrombocytopenia (≤ 30 × 10^9^/L).

Variable	Mild to moderate thrombocytopenia		Severe thrombocytopenia		*p*
	Value	*N*	Value	*N*	
Leukocyte count (10^9^/L)	3.5 (2.5–4.8)	84	3.7 (2.9–6.4)	25	0.339
Leukopenia, *n* (%)	32 (38.1)	84	6 (24.0)	25	0.194
Neutrophil count (10^9^/L)	2.2 (1.6–3.6)	84	2.5 (1.9–4.6)	25	0.261
Neutropenia, *n* (%)	12 (14.3)	84	2 (8.0)	25	0.628
Lymphocyte count (10^9^/L)	0.7 (0.5–1.1)	84	0.8 (0.6–1.4)	25	0.231
Lymphopenia, *n* (%)	46 (54.8)	84	11 (44.0)	25	0.344
Hemoglobin (g/L), mean ± SD	97.4 ± 24.1	84	90.0 ± 26.3	25	0.189
Anemia^a^, *n* (%)	56 (66.7)	84	18 (72.0)	25	0.616
C‐reactive protein (mg/L)	5.9 (4.9–10.5)	84	6.2 (5.1–10.5)	25	0.925
Erythrocyte sedimentation rate (mm/H)	30.0 (14.0–50.0)	83	24.0 (8.0−68.0)	23	0.693
Antinuclear antibody titre	320 (320–1000)	84	320 (320–1000)	25	0.852
Anti‐dsDNA, *n* (%)	46 (54.8)	84	15 (60.0)	25	0.643
Anti‐Smith, *n* (%)	26 (31.0)	84	7 (28.0)	25	0.778
Anti‐U1RNP, *n* (%)	30 (35.7)	84	7 (28.0)	25	0.475
Anti‐ribosomal P protein, *n* (%)	20 (23.8)	84	2 (8.0)	25	0.084
Anti‐Ro60, *n* (%)	38 (45.2)	84	15 (60.0)	25	0.195
Anti‐SSB, *n* (%)	13 (15.5)	84	2 (8.0)	25	0.534
Anti‐Ro52, *n* (%)	32 (38.1)	84	12 (48.0)	25	0.376
Anti‐centromere protein B, *n* (%)	4 (4.8)	84	1 (4.0)	25	1.000
Antiphospholipid antibodies, *n* (%)	8 (16.0)	50	3 (16.7)	18	1.000
Anticardiolipin	7 (14.0)	50	3 (16.7)	18	1.000
Anti–β2‐glycoprotein I	2 (4.0)	50	2 (11.1)	18	0.606
Direct Coombs' test, *n* (%)	7 (21.9)	32	7 (43.8)	16	0.217
Complement 3 (g/L)	0.455 (0.283–0.646)	84	0.448 (0.360–0.740)	25	0.554
Complement 4 (g/L)	0.077 (0.060–0.140)	84	0.080 (0.060–0.118)	25	0.734
Fever, *n* (%)	29 (34.5)	84	7 (28.0)	25	0.543
Rash, *n* (%)	41 (48.8)	84	9 (36.0)	25	0.259
Alopecia, *n* (%)	23 (27.4)	84	9 (36.0)	25	0.406
Oral ulcers, *n* (%)	14 (16.7)	84	2 (8.0)	25	0.451
Arthritis, *n* (%)	13 (15.5)	84	4 (16.0)	25	1.000
Myositis, *n* (%)	2 (2.4)	84	1 (4.0)	25	1.000
Vasculitis, *n* (%)	13 (15.5)	84	8 (32.0)	25	0.121
Pleurisy, *n* (%)	13 (15.5)	84	3 (12.0)	25	0.913
Pericarditis, *n* (%)	11 (13.1)	84	3 (12.0)	25	1.000
Lupus nephritis, *n* (%)	57 (67.9)	84	18 (72.0)	25	0.695
Neurologic involvement, *n* (%)	10 (11.9)	84	3 (12.0)	25	1.000
Cardiac involvement, *n* (%)	7 (8.3)	84	2 (8.0)	25	0.460
Interstitial lung disease, *n* (%)	2 (2.38)	84	4 (16.0)	25	0.034
SLEDAI 2000 score	15.5 (10.0–22.0)	84	17.0 (10.0–25.0)	25	0.715

*Note:* Except where indicated otherwise, values are median (*P*_25_, *P*_75_).

Abbreviations: SLE, systemic lupus erythematosus; SLEDAI 2000, systemic lupus erythematosus disease activity index 2000.

a Anemia was defined as a hemoglobin level < 110 g/L for females or < 120 g/L for males, regardless of etiology.

## 4 Discussion

Thrombocytopenia is a common clinical manifestation of SLE, and its risk factors and treatment have been extensively studied [3, 6, 7, 16]. This study investigated the clinical significance of platelets in SLE. We found that platelet count correlated with indicators of disease activity in SLE, particularly in patients without thrombocytopenia.

Platelets are enucleated subcellular particles derived from megakaryocytes and are the most common blood component after erythrocytes. Platelets patrol the circulatory system and participate in the physiological processes of hemostasis and coagulation by forming aggregations to seal damaged areas of blood vessel walls. There is now growing evidence that platelets are involved in the pathological processes of inflammation and immunity [8]. The serum of patients with SLE contains high levels of immune complexes that activate platelets via recognition of the immunoglobulin receptor FcγRIIA, a process that has been described as a major driver of platelet activation in patients with SLE [9, 10]. Melki et al. reported that platelets released mitochondrial DNA via FcγRIIA upon stimulation with immune complexes in patients with SLE and mouse models [18]. Viral infections, which have been shown to be involved in SLE pathogenesis, promote the exposure of P‐selectin and CD40L (two hallmarks of platelet activation) on platelets via TLR‐7 [11]. Activated platelets induce NETosis (a unique form of neutrophil death) through the interaction of P‐selectin with P‐selectin glycoprotein ligand‐1 expressed on neutrophils [19]. Furthermore, activated platelets may increase the sensitivity of plasmacytoid dendritic cells to interferogenic stimuli via the CD40L–CD40 axis and influence myeloid dendritic cell maturation to regulate adaptive immune responses via CD40L [9, 20]. Our findings of the correlation of platelet counts in patients with SLE with a range of clinical manifestations and disease activity parameters support the theory of platelet involvement in SLE pathogenesis, as reported in the aforementioned basic studies.

Scherlinger et al. believed that activated platelets were more likely to be excreted from the circulation and that thrombocytopenia was, therefore, an indirect potential marker of platelet activation [11]. A recent study revealed that platelets from patients with SLE (SLEDAI: 3.31 ± 4.14) exposed both P‐selectin and the activated form of fibrinogen binding receptor α2bβ3, highlighting platelet activation in patients with SLE despite good control of disease activity [18]. The present study found that even in patients with SLE without thrombocytopenia, there was still a correlation between platelet count and the clinical manifestations of SLE, as well as indicators of disease activity (C3 and C4). Additionally, the platelet count in patients with SLE without thrombocytopenia was significantly lower compared with the healthy controls. Therefore, even in patients with SLE without thrombocytopenia, platelets remain activated and are involved in SLE pathogenesis. Thus, dynamic changes in platelet counts are more clinically relevant in suggesting disease activity in SLE than previously thought.

Compared with patients with SLE without thrombocytopenia, patients with SLE with thrombocytopenia showed more severe disease activity, as reflected in clinical manifestations, complement levels, and SLEDAI 2000 score, which was in general agreement with the Chinese SLE Treatment and Research group (CSTAR) study [16]. The CSTAR study included 2104 patients with SLE, of whom 342 presented with thrombocytopenia and were shown to have a higher prevalence of leukopenia, fever, myositis, nephritis, pleuritis, vasculitis, mucocutaneous lesions, neuropsychiatric lupus, and hypocomplementemia, as well as a significantly higher SLEDAI scores [16]. Furthermore, another study on a large sample of patients with SLE reported a higher incidence of leukopenia, pleurisy, pericarditis, urinary casts, hematuria, pyuria, oral mucosal ulceration, and neurological manifestations and higher disease activity in the thrombocytopenia group (*n* = 804) compared with the nonthrombocytopenia group (*n* = 2336) [21]. Therefore, patients with SLE with thrombocytopenia have a higher level of organ involvement and higher disease activity than patients with SLE without thrombocytopenia, implying that patients with SLE with thrombocytopenia and patients with SLE without thrombocytopenia may represent two distinct groups in SLE. The correlation of platelet counts with hemoglobin levels and a positive direct Coombs' test in patients with thrombocytopenia suggests that more autoantibodies are present and involved in blood cell destruction in this group. Therefore, the decreased platelet count in patients with SLE with thrombocytopenia may be due to not only increased platelet activation but also antiplatelet antibody‐mediated destruction.

When using a platelet count of 50 × 10^9^/L as the cutoff point, there were no significant statistical differences between the clinical characteristics of the mild to moderate and severe thrombocytopenia groups. When the cutoff point was changed to 30 × 10^9^/L, only the prevalence of interstitial lung disease differed between the two groups. However, since the study sample was small, the findings and conclusions may be biased. In a study on 230 patients with SLE‐associated thrombocytopenia, there was no significant difference in clinical or laboratory findings among the groups according to thrombocytopenia severity [22]. In the CSTAR study, the differences between patients with SLE with mild to moderate thrombocytopenia and patients with SLE with severe thrombocytopenia were significantly smaller than those between patients with and without thrombocytopenia [16]. Therefore, patients with SLE‐associated thrombocytopenia may be a relatively homogeneous group.

This study has several shortcomings that should not be overlooked. First, this was a retrospective study, and data were inevitably missing. Second, since this was a single‐center study with a small sample size, the results may be biased. Third, this study did not consider the effect of treatment on platelets, which may have affected the results to some extent. Fourth, the generalisability and validity of the results of the present study were limited by the fact that all patients in this study were hospitalised, and these patients tended to have more severe manifestations.

To summarize, the present study demonstrated that patients with SLE can be categorized into two groups with different clinical features according to the presence or absence of thrombocytopenia. Furthermore, platelet count was correlated with other blood cell counts and complement levels, particularly in patients with SLE without thrombocytopenia, providing clinical confirmation that platelets were not only victims but also active participants in the pathologic progression of SLE. Therefore, an effective treatment for SLE may be the blocking of platelet activation.

## Author Contributions

**Jinlu Ma:** data curation, formal analysis, investigation, software, visualization, writing – original draft. **Lin Zhang:** software, formal analysis. **Mengxue Yan:** investigation, validation. **Zhichun Liu:** conceptualization, supervision, resources. **Leixi Xue:** conceptualization, funding acquisition, methodology, project administration, writing – review and editing.

## Ethics Statement

This study complied with the principles of the World Medical Association Declaration of Helsinki, and was approved by the Ethics Committee of the Second Affiliated Hospital of Soochow University (Approval No. JD‐HG‐2023‐105). Informed consent was not required from patients, which was approved by the Human Ethics Review Committee because the study was based on the participants' previous medical records.

## Conflicts of Interest

The authors declare no conflicts of interest.

## Supporting information

Supporting Tables‐ Clean version.

## Data Availability

The data that support the findings of this study are available from the corresponding author upon reasonable request.
