# Supplementary material for: The Relationship of Platelets With the Clinical Manifestations and Serologic Markers in Systemic Lupus Erythematosus: A Single‐Center Retrospective Study
Source: Immun Inflamm Dis. 2025 Apr 23;13(4):e70201. doi: 10.1002/iid3.70201 (PMC12015639; doi:10.1002/iid3.70201)
Supplement: Supplementary file 1 — Supporting Tables‐ Clean version. [file IID3-13-e70201-s001.docx]

**Supplementary Table 1.** Baseline characteristics of the 418 patients with SLE in our study

| **Variable** | **Value** | **N** |
| --- | --- | --- |
| Female, n (%) | 374 (89.5) | 418 |
| Age (years) | 35.0 (27.0–48.0) | 418 |
| Disease duration (months) | 12.0 (2.0–72.0) | 418 |
| Leukocyte count (10^9^/L) | 4.3 (3.1–6.5) | 418 |
| Leukopenia, n (%) | 90 (21.5) | 418 |
| Neutrophil count (10^9^/L) | 2.8 (1.9–4.7) | 418 |
| Neutropenia, n (%) | 40 (9.6) | 418 |
| Lymphocyte count (10^9^/L) | 1.0 (0.6–1.4) | 418 |
| Lymphopenia, n (%) | 153 (36.6) | 418 |
| Hemoglobin (g/L) | 109 (92–124) | 418 |
| Anemia*, n (%) | 214 (51.2) | 418 |
| Platelet counts (10^9^ /L) | 165.5 (97–218.3) | 418 |
| Thrombocytopenia | 109 (26.1) | 418 |
| C-reactive protein (mg/L) | 5.9 (5.2–9.1) | 418 |
| Erythrocyte sedimentation rate (mm/H) | 32 (14–52) | 404 |
| Antinuclear antibody titre | 1000 (320–1000) | 407 |
| Anti-dsDNA, n (%) | 247 (59.8) | 413 |
| Anti-Smith, n (%) | 140 (34.3) | 408 |
| Anti-U1RNP, n (%) | 168 (41.3) | 407 |
| Anti-ribosomal P protein, n (%) | 97 (23.8) | 407 |
| Anti-Ro60, n (%) | 247 (60.7) | 407 |
| Anti-SSB, n (%) | 65 (16.0) | 407 |
| Anti-Ro52, n (%) | 212 (52.1) | 407 |
| Anti-centromere protein B, n (%) | 11 (2.7) | 407 |
| Antiphospholipid antibodies, n (%) | 43 (18.5) | 232 |
| Anticardiolipin | 40 (17.2) | 232 |
| Anti–β2-glycoprotein I | 13 (5.6) | 232 |
| Direct Coombs’ test, n (%) | 32 (25.8) | 124 |
| Complement 3 (g/L) | 0.550 (0.380–0.775) | 413 |
| Complement 4 (g/L) | 0.099 (0.064–0.154) | 413 |
| Fever, n (%) | 100 (23.9) | 418 |
| Rash, n (%) | 184 (44.0) | 418 |
| Alopecia, n (%) | 137 (32.8) | 418 |
| Oral ulcers, n (%) | 51 (12.2) | 418 |
| Arthritis, n (%) | 77 (18.4) | 418 |
| Myositis, n (%) | 11 (2.6) | 418 |
| Vasculitis, n (%) | 78 (18.7) | 418 |
| Pleurisy, n (%) | 60 (14.4) | 418 |
| Pericarditis, n (%) | 51 (12.2) | 418 |
| Lupus nephritis, n (%) | 264 (63.5) | 416 |
| Neurologic involvement, n (%) | 29 (6.9) | 418 |
| Cardiac involvement, n (%) | 30 (7.2) | 418 |
| Interstitial lung disease, n (%) | 22 (5.3) | 418 |
| SLEDAI 2000 score | 13.0 (8.0–19.0) | 410 |
| Glucocorticosteroid dose, n (%) |  |  |
| Free | 7 (1.7) | 418 |
| Low | 72 (17.2) | 418 |
| Moderate | 114 (27.3) | 418 |
| High | 196 (46.9) | 418 |
| Intravenous puls | 29 (6.9) | 418 |
| Gammaglobulin, n (%) | 41 (9.8) | 418 |

Except where indicated otherwise, values are median (*P*_25_, *P*_75_).

SLE, systemic lupus erythematosus; SLEDAI 2000, systemic lupus erythematosus disease activity index 2000.

*Anemia was defined as a hemoglobin level <110 g/L for females or <120 g/L for males, regardless of etiology.

**Supplementary Table 2.** Comparison of the clinical characteristics of patients with SLE with mild to moderate thrombocytopenia (50–100 × 10^9^/L) and patients with SLE with severe thrombocytopenia (<50 × 10^9^/L)

| **Variable** | **Mild to moderate thrombocytopenia** | | **Severe thrombocytopenia** | | ***P*** |
| --- | --- | --- | --- | --- | --- |
|  | **Value** | **N** | **Value** | **N** |  |
| Leukocyte count (10^9^/L) | 3.5(2.6–6.1) | 66 | 3.6 (2.6–5.2) | 43 | 0.814 |
| Leukopenia, n (%) | 24 (36.4) | 66 | 14(32.6) | 43 | 0.684 |
| Neutrophil count (10^9^/L) | 2.2 (1.7–4.0) | 66 | 2.5 (1.6–4.1) | 43 | 0.901 |
| Neutropenia, n (%) | 6(9.1) | 66 | 8 (18.6) | 43 | 0.147 |
| Lymphocyte count (10^9^/L) | 0.7 (0.5–1.2) | 66 | 0.8 (0.6–1.2) | 43 | 0.399 |
| Lymphopenia, n (%) | 36(54.5) | 66 | 21 (48.8) | 43 | 0.560 |
| Hemoglobin (g/L), mean ± SD | 97.9 ± 25.5 | 66 | 92.1 ± 23.8 | 43 | 0.299 |
| Anemia*, n (%) | 44 (66.7) | 66 | 30 (69.8) | 43 | 0.735 |
| C-reactive protein (mg/L) | 6.5 (5.1–11.2) | 66 | 5.8 (3.7–8.2) | 43 | 0.178 |
| Erythrocyte sedimentation rate (mm/H) | 30.0 (13.0–50.5) | 66 | 28.0 (13.5–51.5) | 43 | 0.982 |
| Antinuclear antibody titre | 320 (100–1000) | 66 | 320 (320–1000) | 43 | 0.374 |
| Anti-dsDNA, n (%) | 36 (54.5) | 66 | 25 (58.1) | 43 | 0.712 |
| Anti-Smith, n (%) | 17 (25.8) | 66 | 16 (37.2) | 43 | 0.203 |
| Anti-U1RNP, n (%) | 24 (36.4) | 66 | 13 (30.2) | 43 | 0.509 |
| Anti-ribosomal P protein, n (%) | 16 (24.2) | 66 | 6 (14.0) | 43 | 0.191 |
| Anti-Ro60, n (%) | 28 (42.4) | 66 | 25 (58.1) | 43 | 0.109 |
| Anti-SSB, n (%) | 10 (15.2) | 66 | 5 (11.6) | 43 | 0.602 |
| Anti-Ro52, n (%) | 23 (34.8) | 66 | 21 (48.8) | 43 | 0.146 |
| Anti-centromere protein B, n (%) | 2 (3.0) | 66 | 3 (7.0) | 43 | 0.621 |
| Antiphospholipid antibodies, n (%) | 5 (13.5) | 37 | 6 (19.4) | 31 | 0.515 |
| Anticardiolipin | 5 (13.5) | 37 | 5 (16.1) | 31 | 1.000 |
| Anti–β2-glycoprotein I | 1 (2.7) | 37 | 3 (9.7) | 31 | 0.484 |
| Direct Coombs’ test, n (%) | 5 (20.0) | 66 | 9 (39.1) | 43 | 0.145 |
| Complement 3 (g/L) | 0.440 (0.274–0.644) | 66 | 0.439 (0.354–0.693) | 43 | 0.525 |
| Complement 4 (g/L) | 0.069 (0.060–0.143) | 66 | 0.080 (0.060–0.124) | 43 | 0.596 |
| Fever, n (%) | 24 (36.4) | 66 | 12 (27.9) | 43 | 0.359 |
| Rash, n (%) | 30 (45.5) | 66 | 20 (46.5) | 43 | 0.914 |
| Alopecia, n (%) | 17 (25.8) | 66 | 15 (34.9) | 43 | 0.307 |
| Oral ulcers, n (%) | 11 (16.7) | 66 | 5 (11.6) | 43 | 0.468 |
| Arthritis, n (%) | 10 (15.2) | 66 | 7 (16.3) | 43 | 0.874 |
| Myositis, n (%) | 2 (3.0) | 66 | 1 (2.3) | 43 | 1.000 |
| Vasculitis, n (%) | 12 (18.2) | 66 | 9 (20.9) | 43 | 0.722 |
| Pleurisy, n (%) | 11 (16.7) | 66 | 5 (11.6) | 43 | 0.468 |
| Pericarditis, n (%) | 7 (10.6) | 66 | 7 (16.3) | 43 | 0.387 |
| Lupus nephritis, n (%) | 46 (69.7) | 66 | 29 (67.4) | 43 | 0.804 |
| Neurologic involvement, n (%) | 9 (13.6) | 66 | 4 (9.3) | 43 | 0.495 |
| Cardiac involvement, n (%) | 7 (10.6) | 66 | 2(4.7) | 43 | 0.454 |
| Interstitial lung disease, n (%) | 2 (3.0) | 66 | 4 (9.3) | 43 | 0.330 |
| SLEDAI 2000 score | 16.0 (9.5–25.0) | 66 | 15.0（10.5–22.0） | 43 | 0.768 |

SLE, systemic lupus erythematosus; SLEDAI 2000, systemic lupus erythematosus disease activity index 2000.

*Anemia was defined as a hemoglobin level <110 g/L for females or <120 g/L for males, regardless of etiology.
